# Assessment of radioactivity and radiological hazards associated with bricks in eastern Nepal

**DOI:** 10.1016/j.heliyon.2024.e24844

**Published:** 2024-01-17

**Authors:** Arun Kumar Shrestha, Ganesh Kumar Shrestha, Buddha Ram Shah, Ram Prasad Koirala

**Affiliations:** a Damak Multiple Campus, Tribhuvan University, Nepal; b Central Department of Physics, Tribhuvan University, Nepal; c Pulchowk Campus, Tribhuvan University, Nepal; d Nepal Academy of Science and Technology, Nepal; e Mahendra Morang Adarsh Multiple Campus, Tribhuvan University, Nepal

**Keywords:** Radionuclides, Activity concentration, Gamma spectrometer, Brick material, Radiological risks

## Abstract

This comprehensive study examines the concentration of natural radionuclides in bricks from the Terai region of the Koshi province, aiming to assess the radioactivity levels and associated radiological hazards, ultimately quantifying the dose limit. The average concentrations of ^226^Ra, ^232^Th, and ^40^K were 27.1 ± 5.7, 42.6 ± 9.8, and 601.5 ± 93.8 Bq/kg, with their respective contributions to total activity being 20.18 %, 45.35 %, 34.48 %,. Despite this, their concentration distribution followed the pattern ^40^K > ^232^Th > ^226^Ra. The elevated presence of ^40^K in the bricks is attributed to the use of phosphate fertilizers in the soil to enhance crop productivity. Notably, the calculated values of radiological hazard parameters, including radium equivalent activity, absorbed gamma dose, and effective dose, are well below the recommended safety thresholds. Consequently, this study suggests that bricks, when used in substantial quantities, pose no significant radiological risks and are considered safe for use as a building material. The extension of such investigations nationwide is recommended to assess the overall radioactivity levels and establish dose limits.

## 1 Introduction

Naturally occurring radionuclides are present everywhere in the earth's crust and their concentrations rely on the geological condition [1,2]. The existence of these radionuclides in building materials contributes to both internal and external radiation exposure in dwellings. External exposure arises from γ radiation emitted from the ^226^Ra, ^232^Th, and ^40^K, while internal exposure results from α, β, and γ radiation emitted from radon and its progenies [3,4]. Approximately two-thirds of the world population's annual effective dose of 2.4 mSv/y is attributed to internal exposure, with the remaining portion originating from external exposure [5,6]. External exposure has the potential to induce skin cancer through the deposition of alpha particles in the skin. On the other hand, the inhalation and ingestion of radioactive radon and its progenies elevate the risk of developing lung and stomach cancer [[7], [8], [9]].

The demand for bricks in construction in Nepal has experienced a significant surge. It is usually made by pressing damp soil into blocks and subsequently subjecting it to kiln heating until it hardens [10]. In residential structures, over 80 % of the building materials, by volume, consist of bricks, serving both internal and external partitioning purposes [11,12]. If the soil or clay contains natural radionuclides, these nuclides are also present in the bricks. The radiation emitted from them can have hazardous effects if people are exposed to higher doses [13]. Despite the escalating demand for bricks as a primary building material in Nepal, there is a notable absence of additional information regarding radiological hazards. Furthermore, no specific guidelines for building materials have been established to safeguard individuals and the environment. Consequently, this issue has garnered significant attention in recent years.

Lamichhane et al. (2021) and Bhatta et al. (2023) undertook measurements of the natural radioactivity in the soils and sediments of Kathmandu Valley. However, a comprehensive literature review indicates a notable absence of further research in this domain within our nation [14,15]. Hence, the primary objective of this study is to ascertain the levels of radioactivity by examining the concentration of ^226^Ra, ^232^Th, and ^40^K in bricks sourced from eastern Nepal and evaluate the associated radiological health hazards, ultimately quantifying the dose limits. The data generated from this investigation will contribute to the establishment of national standards for the utilization and management of building materials. Additionally, it aligns with the achievement of the United Nations’ sustainable development goals, particularly SDGs 8, 11, and 15 by promoting the responsible use of local building materials [16].

## 2 Material and methods

### 2.1 Geological study

Nepal is a small Himalayan country whose more than 83 % of the total area is covered by hills and mountains and the rest of the area is occupied by plain land. The mountains in the Himalayas are active and fragile due to tectonic movement as a result, earthquakes frequently happen in Nepal [17]. The entire territory of Nepal is classified into five different geological zones: (a) Tibetan Himalayan zones (Tethys Himalaya) (b) Greater Himalayan (c) Lesser Himalayan (Mahabharat Mountain Range) (d) Sub Himalayan (Chure or Siwalik), and (e) Gangatic Plain (Terai) [18]. Tibetan-Tethys zone is a rain shadow region and remains dry throughout the year. It is usually made up of sedimentary rocks such as limestone, shale, and sandstone of Precambrian earth. The Greater Himalayan range comprises high-quality metamorphic rocks such as gneisses, migmatites, schists, quartzites, and marbles. Lesser Himalayan mostly contains sedimentary, metasedimentary, and unfossiliferous rocks such as quartzite, slate, phyllite, dolomite, schist, and limestone whereas the Sub Himalayan range mainly consists of sandstone, mudstone, shale, and conglomerate. Gangatic Plain is situated in the southern part and has a thick layer of alluvial sediments. The research site encompasses the plain region which receives fertile soils transported from hilly and mountainous regions, leading to the accumulation of predominantly loamy soil types, including sandy loam, clay loam, and silty loam. These soil characteristics, with their strong binding capacity, make them suitable for clay brick production [19].

### 2.2 Sample collection and preparation

**Fig. 1:**
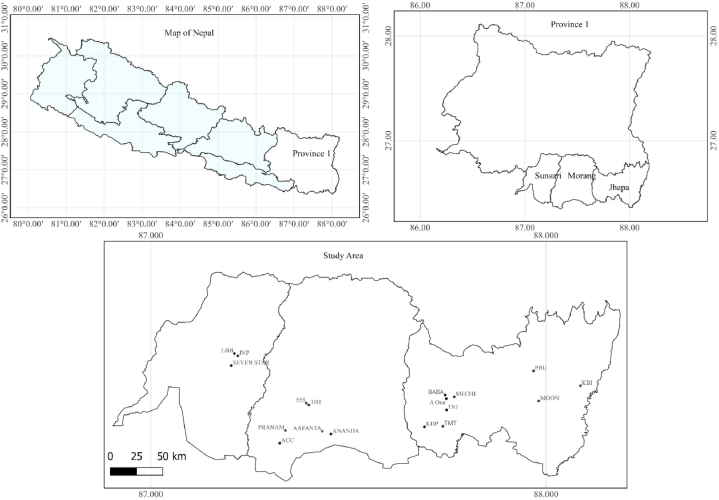
Location of the brick factories in the study area.

In the present study, we collected a total of eighteen samples from three districts (Jhapa, Morang, and Sunsari) of the Koshi Province (Province 1). The brick factory locations, identified by their brand names, are depicted in Fig. 1. Following the standard procedure for preparing the sample mentioned in references [[20], [21], [22]], bricks were crushed into powder and collected separately after passing through a 1 mm mesh sieve. Each sample was dried at 110^O^C in an oven for 24 h to remove the moisture thoroughly. Next, each sample of 1 kg was sealed individually in a Marinelli beaker and stored for more than four weeks to obtain a secular equilibrium between ^226^Ra and ^232^Th and their daughter products.

### 2.3 Activity concentration measurements

For the measurement of activity concentration, a gamma spectrometer coupled with a $3^{\prime \prime }\times 3^{\prime \prime }$ NaI (Tl) scintillation detector from ORTEC and a 14-pin digiBASE photo-multiplier tube, operated using Gamma Vision software, was employed. The energy calibration was performed utilizing ^152^Eu, featuring peak energies at 121.8 keV, 244.7 keV, 444.0 keV, 778.9 keV, 1112.1 keV, and 1408.0 keV. It consists of a first-degree polynomial equation describing the energy dependency on channel numbers in the spectrum [23]. To ensure calibration accuracy, a^60^Co source was employed for regular checks, manifesting two energy peaks at 1332.5 keV and 1173.2 keV. The detector is vertically enclosed in a massive lead shield of cylindrical shape, serving to block extraneous cosmic and background radiation emanating from building materials.

The activity concentration of ^226^Ra was determined using the 351.9 keV (35.8 %) γ line from ^214^Pb and the 609.3 keV (44.8 %) from ^214^Bi [24]. For ^232^Th, it was determined utilizing 583.2 keV (84.5 %) from ^208^Tl, 911.2 keV (26.6 %) from ^228^Ac, and 238.6 keV (43.3 %) from ^212^Pb peaks [25,26]. Additionally, the activity concentration of ^40^K was determined by its gamma line from the energy peak of 1461 keV (10.7 %). The emission probability of gamma disintegration is denoted by the value enclosed in parentheses following the γ-ray energy. Gamma spectra, acquired over a duration of 18,000 s, were stored on the computer. The concentrations of both ^226^Ra and ^232^Th were calculated using average weighted values obtained from their daughter products [27].

Statistical analysis, including descriptive statistics for illustrating the spatial distribution of data and a correlation matrix to establish relationships between these radionuclides and radium-equivalent activity, was conducted. Origin 8 software was employed for both statistical analyses.

### 2.4 Radium equivalent activity (Ra_eq_)

The total activity of Ra226, Th,232 and K40 is characterized using the following relation developed by Beretka and Mathew, (1985) [28]. It is based on the assumption that 1 kg of ^226^Ra, 0.7 kg of ^232^Th, and 13 kg of ^40^K deliver equal gamma dose [29].

(1) $${Ra}_{eq}=A_{Ra}+1.43A_{Th}+0.077A_{K}$$

where, the activity concentration of Ra226, Th,232 and K40 are represented by $A_{Ra},A_{Th},\text{and}\;A_{K}$, respectively.

### 2.5 Absorbed gamma dose radiation ($D_{\gamma }$)

This index assesses the total absorbed dose resulting from the activity concentrations of ^226^Ra, ^232^Th, and ^40^K at 1 m above the ground disregarding the presence of other radionuclides such as ^90^Sr, ^137^Cs, and ^235^U [30,31]. The dose rate ($D_{\gamma }$) is calculated using the formula:

(2) $$D_{\gamma }\left(nGy/h\right)={0.462A}_{Ra}+0.604A_{Th}+0.0417A_{K}$$

Here, 0.462, 0.604, and 0.0417 are the dose conversion factors for radium, thorium, and potassium, respectively.

### 2.6 Annual effective dose equivalent (AED)

The indoor and outdoor annual effective doses resulting from gamma radiation emitted by these radionuclides are computed using the following equations, considering a reference room of dimensions 6 m × 4 m x 3 m with wall density and thickness of wall 2400 kg m^−3^ and 12 cm, respectively [32,33].

(3a) $${\left(\text{AED}\right)}_{in}=D_{\gamma }\left(\text{nGy}/h\right)\times 10^{-6}\times 8760\left(h/y\right)\times 0.80\times 0.7\left(Sv/Gy\right)$$

(3b) $${\left(\text{AED}\right)}_{out}=D_{\gamma }\left(\text{nGy}/h\right)\times 10^{-6}\times 8760\left(h/y\right)\times 0.20\times 0.7\left(Sv/Gy\right)$$

In these equations, $D_{\gamma }$ represents the absorbed dose rate per hour; 8760 represents the number of hours in a year; 0.7 represents the conversion factor (Sv/Gy), and 0.8 and 0.2 are the occupancy factors for indoor and outdoor environments, respectively.

### 2.7 External and internal hazard indices

These indices defined by Krieger (1981) for a model room without doors and windows, with infinitely thick walls, are expressed through the following formulas [34]:

(4a) $$H_{ex}=\frac{A_{Ra}}{370\left(Bq/kg\right)}+\frac{A_{Th}}{259\left(Bq/kg\right)}+\frac{A_{K}}{4810\left(Bq/kg\right)}\leq 1$$

(4b) $$H_{in}=\frac{A_{Ra}}{185\left(Bq/kg\right)}+\frac{A_{Th}}{259\left(Bq/kg\right)}+\frac{A_{K}}{4810\left(Bq/kg\right)}\leq 1$$

These indices are employed to restrict the gamma dose to 1.5 mSv/y, ensuring the safe utilization of construction materials [35].

#### 2.7.1 Gamma index ($I_{\gamma }$)

It is proposed by the European Commission for European standard room and calculated as [36],

(5) $$I_{\gamma }=\frac{A_{Ra}}{300}+\frac{A_{Th}}{200}+\frac{A_{K}}{3000}\leq 1$$

It was developed to restrict the gamma dose to 1 mSv/y.

#### 2.7.2 Alpha index ($I_{\alpha }$)

It assesses the internal hazard due to the inhalation of radon emitted from the construction materials.

(6) $$I_{\alpha }=\frac{A_{Ra}}{200}$$

To ensure the safe utilization of building materials, the calculated values should be less than one. The prescribed and upper thresholds for ^226^Ra concentrations are 100 Bq/kg and 200 Bq/kg respectively [37]. If the radium activity content exceeds 200 Bq/kg, it is anticipated that indoor radon concentrations will surpass 200 Bqm^−3^ [38].

### 2.8 Excess lifetime cancer risk (ELCR)

This index quantifies the potential cancer risk for millions of individuals annually and it is calculated through the relation:

(7) ELCR=AED×DL×RF

Here, the duration of life (DL) for Nepalese people is 70 years and the risk factor (RF) is 0.055 × 10^−3^ according to the guidelines by ICRP (1991) [39].

## 3 Results and discussion

### 3.1 Activity concentrations of ^226^Ra, ^232^Th, and ^40^K in bricks

The activity of each sample was measured twice and Table 1 presents the average values along with the standard deviation. The ratios of ^226^Ra, ^232^Th, and ^40^K are also provided in Table 1 to characterize the soil properties. The activity concentration of ^226^Ra ranged from 19.2 ± 0.8 to 37.7 ± 0.8 Bq/kg, with an average value of 27.1 ± 5.7 Bq/kg. The observed radioactivity was minimum in the A ONE brick and maximum in the AANANDA brick. The activity concentration of ^232^Th varied from 29.9 ± 10.8 to 60.5 ± Bq/kg, with an average value of 42.6 ± 9.8 Bq/kg. The minimum was noted in brick KBI and the maximum in brick LBB. Similarly, the average activity concentration of ^40^K was 601.5 ± 93.8 Bq/kg, ranging from 483 ± 9.3 Bq/kg for BABA bricks to 826.2 ± 31.9 Bq/kg for LBB bricks. According to UNSCEAR (1993), the worldwide average activity for construction materials with ^226^Ra, ^232^Th, and ^40^K is 50, 50, and 500 Bq/kg, respectively [40].

**Table 1:** The measured values of^226^Ra,^232^Th, and^40^K in the brick samples.

SN	Samples	^226^Ra (Bq/kg)	^232^Th (Bq/kg)	^40^K (Bq/kg)	^40^K/^226^Ra	^40^K/^232^Th	^232^Th/^226^Ra
1	A ONE	19.2 ± 0.8	33.8 ± 4.9	507.6 ± 1.1	26.4	15.0	1.8
2	JNP	33.5 ± 1.3	55.7 ± 6.3	670.5 ± 33.2	20.0	12.0	1.7
3	KBI	21.4 ± 0.5	29.9 ± 10.8	560.2 ± 11.9	26.2	18.7	1.4
4	KDP	27 ± 2.8	43.4 ± 1.0	572.5 ± 0.3	21.2	13.2	1.6
5	MECHI	21.7 ± 1.3	32 ± 1.2	524.3 ± 15.1	24.2	16.4	1.5
6	PRANAM	24.3 ± 1.5	33.5 ± 2.3	532.9 ± 16.9	21.9	15.9	1.4
7	SEVEN STAR	19.9 ± 1.8	35.4 ± 3.2	508.5 ± 11.9	25.5	14.4	1.8
8	TBT	33 ± 0.3	53.4 ± 0.0	711.8 ± 19.9	21.6	13.3	1.6
9	555	23.2 ± 2.7	39.9 ± 2.0	540.7 ± 16.9	23.3	13.5	1.7
10	AAFANTA	34.8 ± 3.9	58.8 ± 1.5	666.3 ± 0.6	19.1	11.3	1.7
11	ACC	32 ± 3.0	50.8 ± 12.9	730.5 ± 8.7	22.8	14.4	1.6
12	LBB	34.4 ± 3.5	60.5 ± 4.3	826.2 ± 31.9	24.0	13.7	1.8
13	MOON	23 ± 1.6	40.2 ± 4.2	572.3 ± 12.9	24.9	14.2	1.7
14	PBU	26 ± 0.1	41.3 ± 2.1	593.0 ± 14.6	22.8	14.4	1.6
15	TEJ	26 ± 0.5	30.6 ± 12.9	531.4 ± 9.5	20.4	17.4	1.2
16	TMT	27.6 ± 1.2	41.4 ± 0.6	610.2 ± 24.3	22.1	14.7	1.5
17	AANANDA	37.7 ± 0.8	48.3 ± 7.4	685.2 ± 32.9	18.2	14.2	1.3
18	BABA	24 ± 0.8	38.1 ± 2.8	483.0 ± 9.3	20.1	12.7	1.6
Average		27.1	42.6	601.5	22.5	14.4	1.6
Std. Dev.		5.7	9.8	93.8	2.4	1.8	0.2
Range		19.2–37.7	29.9–60.5	483.0–826.2	18.2–26.4	11.3–18.7	1.2–1.8

In comparison, the average activity in the examined samples was marginally lower in ^226^Ra and ^232^Th, but slightly higher for ^40^K. The average ratio of ^232^Th to ^226^Ra is 1.6, indicating thorium's dominance over radium in the bricks, likely due to the presence of monazite [41,42]. The ratios of ^40^K/^226^Ra and ^40^K/^232^Th were 22.5 and 14.4, respectively, with their relative abundances in the order of ^40^K > ^232^Th > ^226^Ra. The high concentration of ^40^K may be attributed to the existence of potassium feldspar in the samples [43]. Additionally, the excessive use of chemical fertilizers on the lands to enhance crop productivity could contribute to the elevated levels of ^40^K in the samples [44]. Therefore, it is suggested that monazite and feldspar are present in varying proportions in all brick samples.

### 3.2 Pearson's correlation coefficient

**Fig. 2:**
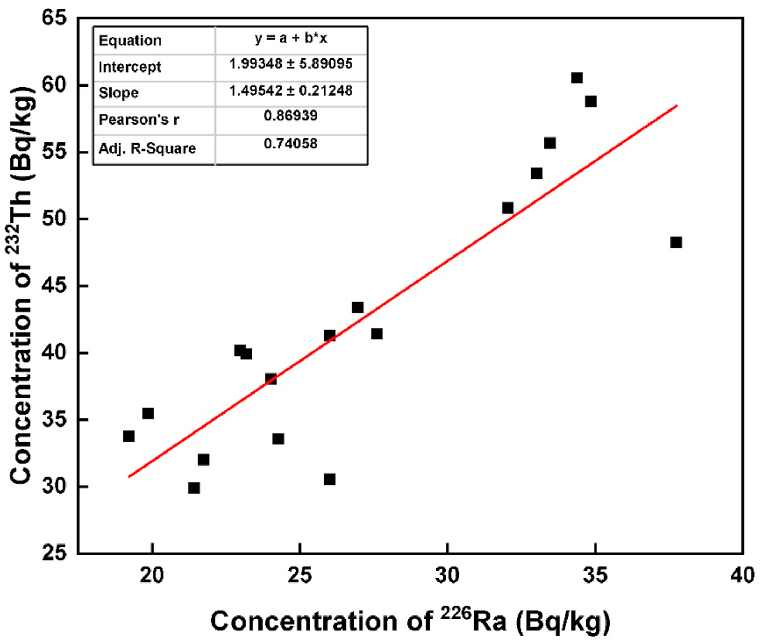
The correlation between the concentration of ^226^Ra and ^232^Th.

**Fig. 3:**
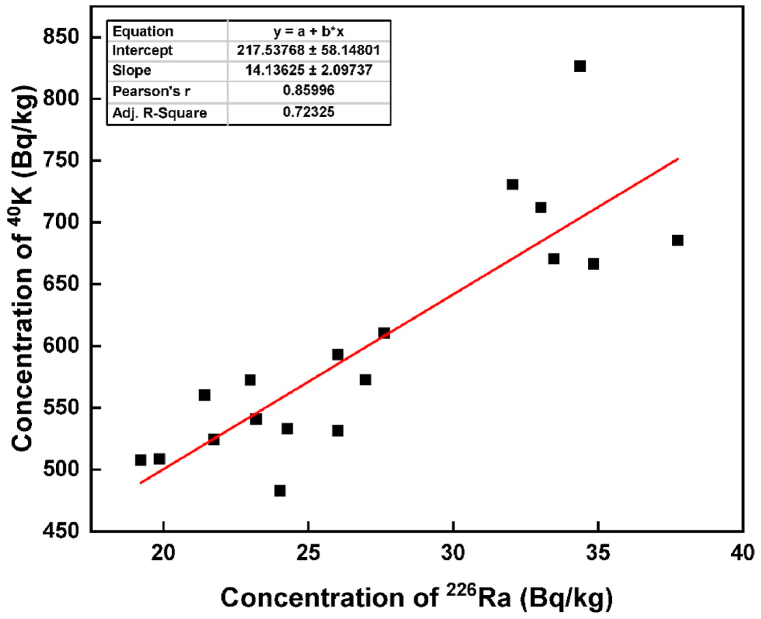
The correlation between the concentration of ^226^Ra and ^40^K.

**Fig. 4:**
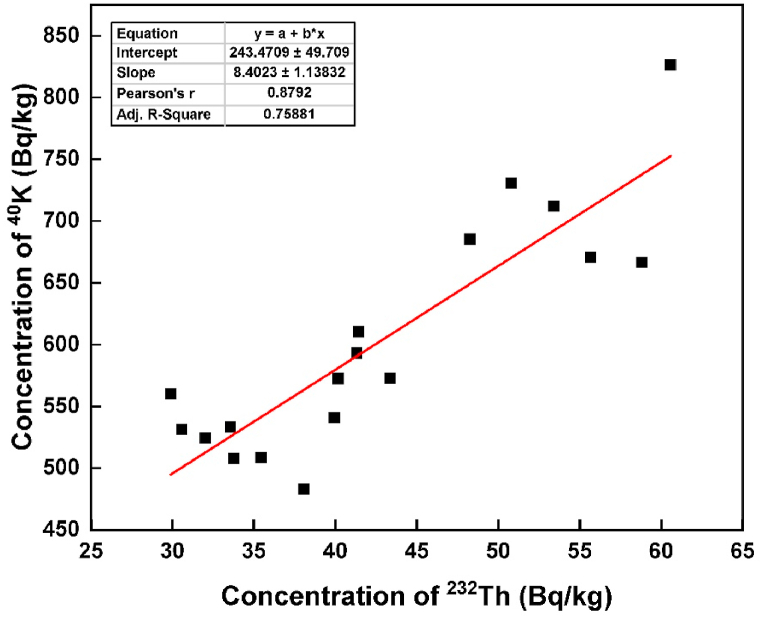
The correlation between the concentration of ^232^Th and ^40^K.

**Fig. 5:**
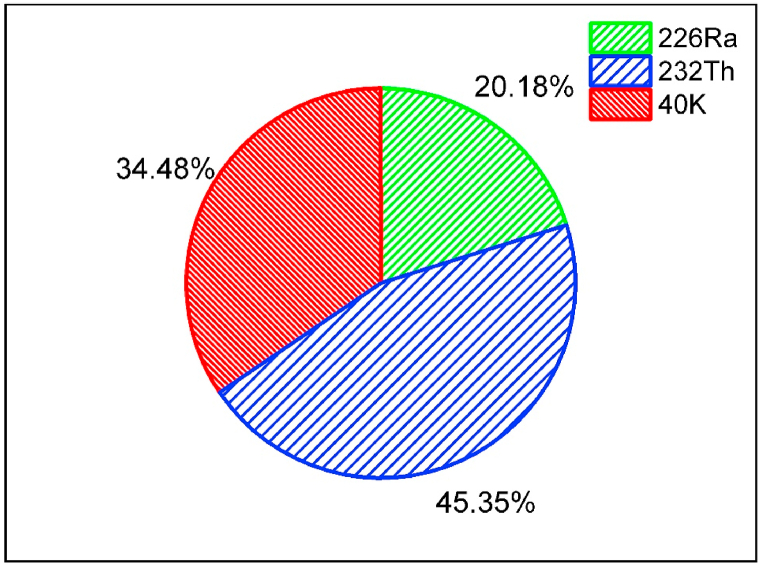
Relative contributions to total activity concentrations due to ^226^Ra, ^232^Th, and ^40^K in brick samples.

The correlation coefficient was employed to explore the linear relationship among ^226^Ra, ^232^Th, and ^40^K in the brick samples, with results summarized in Table 2. A notably strong positive correlation was identified between ^226^Ra and ^232^Th (r=0.869), ^226^Ra and ^40^K (r=0.860), and ^232^Th and ^40^K (r=0.879). All these parameters exhibited significant correlations with Ra_eq_. The robust correlation between ^226^Ra, ^232^Th, and ^40^K is likely attributed to the uniform distribution of radionuclides in the bricks [19]. Subsequently, a scatter diagram was also generated based on the correlation matrix, as illustrated in Fig. 2, Fig. 3, and Fig. 4, respectively. The adj. R-square values were 0.741 for ^226^Ra and ^232^Th, 0.721 for ^226^Ra and ^40^K, and 0.759 for ^232^Th and ^40^K, indicating a high goodness of fit for the data points. This suggests that the model is adept at predicting the values of the dependent variables. The relative contributions of ^226^Ra, ^232^Th, and ^40^K to the total activity concentration are depicted in Fig. 5, with their respective contributions being 45.35 %, 34.48 %, and 20.18 %, respectively. It implies that thorium has a higher contribution compared to uranium and potassium in the overall activity concentration.

**Table 2:** Correlation matrix among the radionuclides and radium equivalent activity.

	^226^Ra	^232^Th	^40^K	Ra_eq_
^226^Ra	1			
^232^Th	0.869	1		
^40^K	0.860	0.879	1	
Ra_eq_	0.932	0.979	0.945	1

### 3.3 Evaluation of radiological hazard parameters

To assess the radiation risks to human health, various radiological parameters, including radium equivalent activity, were computed and summarized in Table 3. The Ra_eq_ estimated using Eq. (1), ranged from 106.6 to 184.5 Bq/kg, with a mean value of 134.4 ± 25.9 Bq/kg. This falls below the limit of 370 Bq/kg recommended for any building materials to maintain an external dosage under 1.5 mSv/y [45]. The measured Ra_eq_ values in the bricks were found to be within the prescribed required limit, indicating that their use as building materials does not pose radiation hazards. For other applications, specific limitations apply: industrial use is restricted to 740 Bq/kg, construction of roads and railroads is to 2200 Bq/kg, and landfill materials are capped at 3700 Bq/kg. Utilizing materials with Ra_eq_ exceeding 3700 Bq/kg is prohibited [46].

**Table 3:** Radiological hazard parameters in brick samples.

SN	Brand of bricks	Ra_eq_ (Bq/kg)	D_y_ (nGy/h)	AED (mSv/y)		Hazard Index		Gamma index (I_y_)	Alpha index (I_α_)	ELCR (10^−3^)
				In	Out	External	Internal			
1	A ONE	106.6	50.4	0.25	0.06	0.29	0.34	0.4	0.10	0.95
2	JNP	164.8	77.1	0.38	0.09	0.44	0.53	0.61	0.17	1.45
3	KBI	107.3	51.3	0.25	0.06	0.29	0.35	0.41	0.11	0.97
4	KDP	133.1	62.6	0.31	0.08	0.36	0.43	0.5	0.13	1.18
5	MECHI	107.8	51.2	0.25	0.06	0.29	0.35	0.41	0.11	0.97
6	PRANAM	113.2	53.7	0.26	0.07	0.31	0.37	0.43	0.12	1.01
7	SEVEN STAR	109.7	51.8	0.25	0.06	0.30	0.35	0.41	0.10	0.98
8	TBT	164.2	77.2	0.38	0.09	0.44	0.53	0.61	0.16	1.46
9	555	121.9	57.4	0.28	0.07	0.33	0.39	0.46	0.12	1.08
10	AAFANTA	170.2	79.4	0.39	0.10	0.46	0.55	0.63	0.17	1.50
11	ACC	160.9	75.9	0.37	0.09	0.43	0.52	0.60	0.16	1.43
12	LBB	184.5	86.9	0.43	0.11	0.50	0.59	0.69	0.17	1.64
13	MOON	124.5	58.8	0.29	0.07	0.34	0.40	0.47	0.11	1.11
14	PBU	130.7	61.7	0.30	0.08	0.35	0.42	0.49	0.13	1.16
15	TEJ	110.7	52.6	0.26	0.06	0.30	0.37	0.42	0.13	0.99
16	TMT	133.8	63.2	0.31	0.08	0.36	0.44	0.50	0.14	1.19
17	AANANDA	159.5	75.2	0.37	0.09	0.43	0.53	0.59	0.19	1.42
18	BABA	115.7	54.2	0.27	0.07	0.31	0.38	0.43	0.12	1.02
Average		134.4	63.4	0.31	0.08	0.36	0.44	0.50	0.14	1.20
Std. Dev.		25.9	12.0	0.06	0.02	0.07	0.08	0.09	0.03	0.23
Range		106.6–184.5	50.4–86.9	0.25–0.43	0.06–0.11	0.29–0.50	0.34–0.59	0.40–0.69	0.10–0.19	0.95–1.64

Further, the absorbed gamma dose $\left(D_{\gamma }\right)\text{,}$ computed using Eq. (2), ranged from 50.4 to 86.9 nGy/h, with an average value of 63.4 ± 12 nGy/h. In the majority of the samples, this value was lower than the global average of 86 nGy/h, except for LBB bricks. However, it was still lower than the Indian average value of 90 nGy/h for building materials [33]. The ${\;AED}_{in}$ and ${\;AED}_{out\;}$ were calculated using Eqs. (3a), (3b), respectively, and are presented in columns 5 and 6 of Table 3. The minimum ${\;AED}_{in\;}$ of 0.25 was recorded in A ONE, KBI, MECHI, and SEVEN STAR bricks, while the maximum value of 0.47 was observed in LBB bricks. The average ${\;AED}_{in\;}$ was 0.31 ± 0.06, which is lower than the worldwide average value of 1 mSv/y for all building materials [22]. Similarly, the average ${\;AED}_{out\;}$ was 0.08 ± 0.02, ranging from a minimum of 0.06 in the various bricks and a maximum of 0.11 in LBB brick.

The H_ex_ and H_in_ were computed using Eq. (4a) and Eq. (4b), ranging from 0.29 to 0.50 and 0.34 to 0.59, with averages of 0.36 ± 0.07 and 0.44 ± 0.08, respectively. Both H_ex_ and H_in_ were found to be less than the critical value of unity, indicating that the bricks are safe for use as building materials. Similarly, the gamma index $\left(I_{\gamma }\right)$, estimated using Eq. (5), ranged from 0.40 to 0.69, with an average value of 0.50 ± 0.09. Since the average value of $I_{\gamma }$ is less than unity, it confirms that the effective dose of gamma radiation delivered by these brick samples is within the safe limit of 1 mSv/y. The alpha index ($I_{\alpha }$), obtained using Eq. (6), varied from 0.10 to 0.19, with an average value of 0.14 ± 0.03. This indicates that indoor radon concentrations did not exceed the prescribed value of 200 Bq/kg, and it remains below the upper limit of 1 for internal exposure [47]. Only when these conditions are met, the bricks are considered safe from internal radiation exposure. The ELCR calculated using Eq. (7) also varied between 0.95 × 10^−3^ to 1.64 × 10^−3^ and its mean value was 1.20 ± 0.23 × 10^−3^, which was less than the global average value of 1.16 × 10^−3^ [48]. It demonstrates that even indoors, there is very little chance of developing cancer from the gamma radiation emitted from construction materials.

Our results have been compared with the average value for three radionuclides and their corresponding Ra_eq_ from 10 different countries, as presented in Table 4. In this comparison, the average activity concentration of ^226^Ra (27.1 Bq/kg) in our study was found to be similar to that of Albania, Iran, Pakistan, Sri Lanka, and Egypt. Several countries, including China, Vietnam, Tunisia, and Turkey, recorded higher levels, while India reported a lower value. The result for ^232^Th (42.6 Bq/kg) in our study was comparable to those of the other countries, except for Sri Lanka (72 Bq/kg). Similarly, our ^40^K result (601.5 Bq/kg) was in line with those from Vietnam, Sri Lanka, Pakistan, Tunisia, and Albania. Egypt and India exhibited lower values compared to the global average, as shown in the table. It's noteworthy that the Ra_eq_ for bricks from all nations, including our study, is less than 370 Bq/kg. This indicates that these bricks do not pose a radiological risk when utilized as construction material for building structures.

**Table 4:** A comparison of the average value of^226^Ra,^232^Th,^40^K, and Ra_eq_ of bricks with other studies worldwide.

Country	No of sample	Ra-226 (Bq/kg)	Th-232 (Bq/kg)	K-40 (Bq/kg)	Ra_eq_ (Bq/kg)	References
India	32	5.9	54.7	377.9	113.2	[49]
Turkey	52	58.6	51.6	829.8	196.3	[50]
Vietnam	6	41.6	54.8	614.6	167.3	[51]
Iran	45	33	30	700	129.8	[52]
Pakistan	140	35	42	624	143.1	[19]
Sri Lanka	24	35	72	585	183.0	[53]
Egypt	19	24.1	24.0	258	78.3	[54]
Albania	9	33.4	42.2	644.1	143.3	[55]
China	14	46	56	846	191.2	[56]
Tunisia	7	46.0	51.3	631.4	168	[57]
Nepal	18	27.1	42.6	601.5	134.3	Present work

## 4 Conclusion

In summary, the average concentrations of ^226^Ra, ^232^Th, and ^40^K in bricks were found to be 27.1 ± 5.7, 42.6 ± 9.8, and 601.5 ± 93.8 Bq/kg, respectively. These values align with worldwide average limits, and the relative contributions of these radionuclides to the total activity are 20.18 %, 45.35 %, and 34.48 %, respectively. The strong correlation observed among these radionuclides suggests a common origin. Notably, the Ra_eq_ value for building materials falls below the standard permissible limit of 370 Bq/kg, and the absorbed dose rate is lower than the worldwide average of 86 nGy/h. The assessment of all radiological hazard parameters indicates that these bricks do not pose significant biological effects to inhabitants. The comparative study further reveals that the average value of these radionuclides is in line with those observed in other countries. Based on the analysis, it can be concluded that these bricks are devoid of any radiological hazard and are safe for use as a building material. Finally, it is recommended to extend this type of work across the country to comprehensively assess the levels of radioactivity in different regions.

## Data availability

All data generated or analyzed during this study are included in this published article.

## Additional information

There is no additional information in this paper.

## CRediT authorship contribution statement

**Arun Kumar Shrestha:** Writing – original draft. **Ganesh Kumar Shrestha:** Writing – review & editing, Supervision. **Buddha Ram Shah:** Writing – review & editing, Supervision, Methodology. **Ram Prasad Koirala:** Writing – review & editing, Supervision.

## Declaration of competing interest

The authors declare the following financial interests/personal relationships which may be considered as potential competing interests:Arun Kumar Shrestha reports University Grants Commission Nepal provided a Ph.D. fellowship and research support grant including a travel reimbursement. If there are other authors, they declare that they have no known competing financial interests or personal relationships that could have appeared to influence the work reported in this paper.
